# A Virtual Electronic Nose for the Efficient Classification and Quantification of Volatile Organic Compounds

**DOI:** 10.3390/s22197340

**Published:** 2022-09-27

**Authors:** Guillem Domènech-Gil, Donatella Puglisi

**Affiliations:** Department of Physics, Chemistry and Biology, Division of Sensor and Actuator Systems, Linköping University, 581 83 Linköping, Sweden

**Keywords:** gas sensors, virtual arrays, volatile organic compounds, selectivity, quantification, machine learning, indoor air quality

## Abstract

Although many chemical gas sensors report high sensitivity towards volatile organic compounds (VOCs), finding selective gas sensing technologies that can classify different VOCs is an ongoing and highly important challenge. By exploiting the synergy between virtual electronic noses and machine learning techniques, we demonstrate the possibility of efficiently discriminating, classifying, and quantifying short-chain oxygenated VOCs in the parts-per-billion concentration range. Several experimental results show a reproducible correlation between the predicted and measured values. A 10-fold cross-validated quadratic support vector machine classifier reports a validation accuracy of 91% for the different gases and concentrations studied. Additionally, a 10-fold cross-validated partial least square regression quantifier can predict their concentrations with coefficients of determination, R^2^, up to 0.99. Our methodology and analysis provide an alternative approach to overcoming the issue of gas sensors’ selectivity, and have the potential to be applied across various areas of science and engineering where it is important to measure gases with high accuracy.

## 1. Introduction

The omnipresence of gases that are harmful to human health and are estimated to cause the premature death of about 7 million people worldwide every year [1] has made chemical gas sensors a topic of extreme importance. There have been undeniable efforts to develop new sensing materials and devices, and to improve their performance. Over the past seven decades, extensive research has been conducted to develop gas sensors with high sensitivity, selectivity, stability, speed of response, and reliability, and low power consumption. Various types of gas sensor technologies, such as photoionization detectors, infrared sensors, metal oxide semiconductor sensors, chemiresistors, and field-effect-based sensors, have been widely investigated and used in real-world applications, including medical, environmental, automotive, industrial, and agricultural applications [2]. However, from early [3,4,5,6,7,8,9,10,11,12,13,14] to more recent developments [15,16,17,18,19,20,21,22,23], the selectivity issue has never been properly overcome.

Electronic noses appear to be promising candidates for selectivity enhancement, thanks to elaborated “fingerprint” patterns produced by a group of robust features that are unique for each gas and concentration studied [24,25,26,27,28,29,30]. By combining manifold features and applying machine learning (ML) techniques, it is possible to identify and differentiate between gases of interest, i.e., to enhance selectivity [31,32,33,34,35]. Unfortunately, despite being a potential pathway to overcoming selectivity problems, multi-sensor electronic noses suffer from high manufacturing costs, complexity, and high levels of power consumption. Here, we propose the use of one single sensor, operating in a dynamic mode (DM), as a virtual sensor array, and demonstrate the power of the synergy between DM operation and ML algorithms as an effective tool to enhance selectivity among similar gases, at the same time as reducing manufacturing costs, hardware complexity, and power consumption. To achieve this goal, we selected three short-chain oxygenated volatile organic compounds (VOCs) that appear sequentially as products and by-products of each other, and are very difficult to discriminate. By implementing specific ML algorithms, we were able to differentiate between formaldehyde (CH_2_O), formic acid (CH_2_O_2_), and acetic acid (CH_3_COOH), and quantify their concentrations. These three VOCs are widely distributed compounds produced on a large scale in industrial reactors, and are used as synthesis reagents in many applications [36,37,38]. Currently, none of the low-cost gas-sensing technologies available on the market can differentiate between and quantify the three VOCs studied in this work. Therefore, the possibility of measuring such gases with high accuracy constitutes a major development for these applications.

By use of a virtual electronic nose coupled with computing power, we demonstrate the possibility of efficiently and cost-effectively discriminating between, classifying, and quantifying similar target gases; thus, this research represents a significant step forward in the development of selective gas sensor technologies. 

## 2. Materials and Methods

### 2.1. Gas Sensors

A gas sensitive silicon-carbide-based field effect transistor (SiCFET) prototype and a digital temperature and humidity sensor, SHTC1, from Sensirion AG (Stäfa, Switzerland) were connected in series to a gas stream and operated simultaneously for all measurements.

The SiCFETs were manufactured from an n-type 4H-SiC substrate (ca. 350 µm thickness and a doping level of ca. 3 × 10^18^ cm^−3^) using the fabrication process described elsewhere [36], and a nanostructured porous iridium (Ir) gate deposited by DC magnetron sputtering at an argon pressure of 50 mTorr to a total thickness of about 30 nm. The chosen catalytic metal promotes the optimal gas sensing mechanisms for the studied gases and temperature range.

The electrical and gas-sensing characterizations were performed using a SiCFETs glued on top of a ceramic heater (Heraeus Sensor-Nite GmbH, Hanau, Germany), and next to a Pt-100 temperature sensor. The heater was used to increase the temperature of the SiCFETs and to promote gas–solid interactions in the gate material, and the temperature sensor was used to control the temperature of the gas sensor during measurements. The SiCFET chip, the heater, and the Pt-100 were mounted on top of a 16-pin TO8 header; they were then spot welded and gold-wire bonded to allow electrical access.

Current–voltage (I–V) measurements were carried out on eight SiCFET devices before testing as gas sensors. The I–V characterization was performed at 300 °C, in synthetic air (80% N_2_, 20% O_2_, at a flow rate of 100 mL/min). All measurements were carried out by sweeping the voltage over the drain-source contacts, V_DS_, from 0 to 5 V at a rate of 0.1 V/s, and measuring the drain current, I_D_. The gate-source voltage, V_GS_, was kept at 0 V. All gas sensor measurements were performed at V_DS_ equal to 4 V, corresponding to the saturation region of the sensor, which is the optimal operating point of the device [39].

### 2.2. Operation of the Sensors and Experimental Setup

The sensors were tested under controlled conditions inside custom-made stainless-steel chambers with a volume of about 3 mL. The gas flow mixtures introduced into the measurement chambers were managed by means of Labview software, which runs a gas-mixing system with six Bronkhorst mass flow controllers (MFCs). To acquire and record the sensor signals during the gas tests, electrical measurements were performed using Python software that was developed to command an analogue front-end controller (3S GmbH, Saarbrücken, Germany), simultaneously supplying a voltage and measuring the current of the SiCFETs, in order to control the heater and to monitor the Pt-100 outcomes.

**Figure 1:**
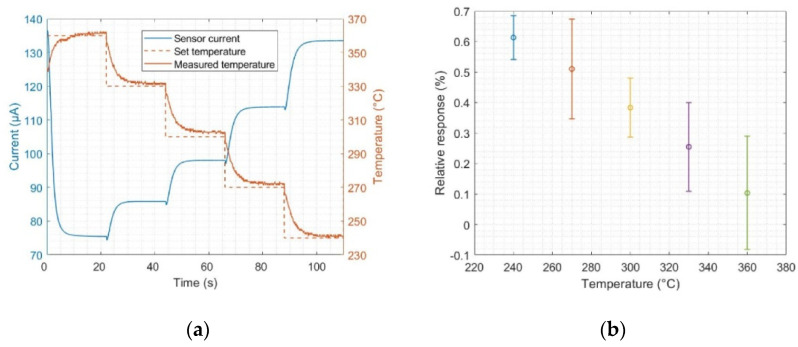
(**a**) Current evolution of a SiC-based field-effect transistor sensor during a temperature cycle between 360 and 240 °C, including the evolution of set and measured temperatures; (**b**) mean values and standard deviations of the relative response, calculated from the virtual sensors at the different studied temperatures for 1 ppm formic acid, as an example. By virtual sensor, we mean the mean value of the sensor signal at each temperature plateau gathered along consecutive cycles to produce an equivalent sensor signal.

During all measurements, the SiCFETs were operated in DM using a temperature-cycled operation between 240 and 360 °C, with five steps of 30 °C each maintained for 22 s, resulting in a total of 110 s per cycle (Figure 1a). The cycles were continuously concatenated during the measurements. Since the data acquisition rate is 10 Hz, each cycle has a total of 1100 data points. The time length of the steps was optimised for proper sensor signal stabilization at each studied temperature while minimizing the total cycle time. A stable signal after each temperature variation is necessary to avoid transient behaviour at the temperature plateaus; additionally, a short cycle opens up the possibility of implementing this type of procedure in daily-life applications that require relatively short response times. The SiCFETs and temperatures studied have been demonstrated to induce ultra-high sensitivity in dry air for different VOCs [40] and a considerable difference in the *relative response* at the temperature plateaus (Figure 1b), which is beneficial for later discrimination, classification, and quantification. For this reason, we used the aforementioned temperature range in our measurements.

The *relative response* of the sensors towards adsorbing species is here defined as the relative change in the sensor’s current as an absolute value, which is expressed as:

(1) $$Relative\;response\;\left(\%\right)=\frac{\left|I_{air}-I_{gas}\right|}{I_{air}}\cdot 100$$

where $I_{air}$ corresponds to the sensor’s current value when exposed to synthetic air (SA), and $I_{gas}$ corresponds to the sensor’s current value when the target gas is introduced into the SA gas flow.

### 2.3. Gas Measurements

The sensors were characterised by exposure to different concentrations of CH_2_O, CH_2_O_2_, and CH_3_COOH, randomly varying between 250 and 3000 parts per billion (ppb), using dry SA as the carrier gas. The total flow over the sensors was kept at a constant flow rate of 100 mL/min. Repetitions of random exposures to the different gas concentrations were carried out, instead of sequential increasing or decreasing concentrations, to avoid memory effects of accumulated adsorbents that could lead to incorrect conclusions. The gas-mixing system was calibrated with the SHTC1 sensor at 20 °C before the SiCFETs were tested. The SHTC1 humidity sensor measured <5% RH under dry SA conditions. Therefore, the measurements presented here under dry SA include less than 5% RH. The gas measurements included: (i) 240 min of exposure to the carrier gas (SA) to allow the device to stabilise the baseline; (ii) 20 min of exposure to the studied gas; and (iii) 40 min in dry SA to recover the baseline after gas exposure.

### 2.4. Data Evaluation

ML techniques were implemented to analyse the results of the acquired data from the SiCFETs working in DM. First, a baseline correction was implemented by smoothing and standardizing the sensor signal. The smoothing step was implemented to remove as much noise as possible and, at the same time, preserve the sensor signal change when exposed to the gases of interest. For this purpose, a Savitzky–Golay filter was used [41]. The standardization applied here to reduce sensor drift from both the baseline and the response of the sensor [42] was auto-scaling, which can be expressed as:

(2) $$y_{ij}^{stand}=\frac{y_{ij}-\bar{y_{i}}}{\sqrt{\frac{1}{n-1}\sum_{j-1}^{n}{\left(y_{j}-\bar{y_{i}}\right)}^{2}}}\cdot 100$$

where $y_{ij}$ and $y_{ij}^{stand}$ are the raw and standardised data values *j* in a cycle *i*, $\bar{y_{i}}$ is the mean value of the data values in the *i*^th^ cycle, $y_{j}$ is the data values of a certain cycle, and *n* is the total number of points in one cycle.

**Figure 2:**
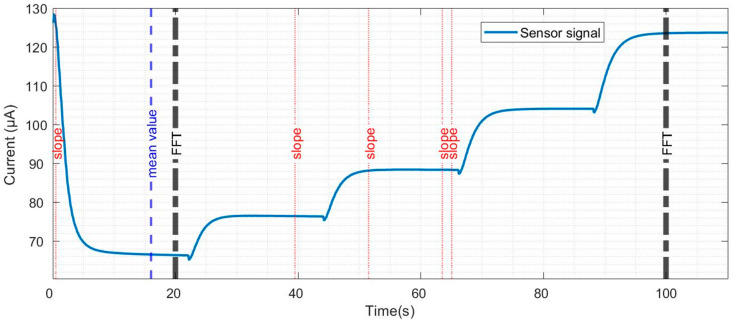
Current evolution of a SiC-based field-effect transistor sensor during a temperature cycle, and the features selected for pattern recognition.

Feature selection and extraction are some of the most important steps for data evaluation. This process is related to each gas or set of gases and the concentrations being studied and, therefore, needs to be customised on a case-by-case basis to find the parameters that best highlight the particularities in the sensor signals for the different exposures under evaluation. In general, many features are extracted, and, among these, the most relevant ones are considered for gas discrimination, classification, and/or quantification [34,35,43,44,45]. This process is called a top-down dimensionality reduction and is the second step we implemented in our data evaluation process. The features calculated in each cycle from the sensor signal were: (i) 55 mean values calculated every two seconds, (ii) 220 slopes calculated every 0.5 s, (iii) 22 Fast Fourier Transforms (FFT) calculated every five seconds, (iv) one integral of the area under the sensor signal of each cycle, and (v) one lifting of each cycle. The lifting is the difference between the first and last points in the cycle. The total number of features evaluated was 299. The features were evaluated using a sequential forward selection (SFS) method and eight features were selected. The selected features (Figure 2) are one mean value, five slopes, and two FFT values.

Classification was performed by implementing LDA [43,46] and 10-fold CV SVM, which classifies the data by finding the best hyperplane that separates the different defined groups [47]. To obtain a more robust, stable, and reliable model, all concentrations evaluated for each studied gas were included in each defined group [48]. 

PLSR was used as quantification technique [49,50]. To improve the reliability of the PLSR model, we implemented a 10-fold CV [50]. To test the stability of the trained model and to ensure a robust and stable model, the data were randomly split into two groups of data sets: the training set and the test data [50]. The training data subset randomly selected 80% of the data, leaving 20% of the data for the test subset. In this way, the test data were classified based on the model established with the training data set. Overfitting was avoided by always keeping the number of exposure cycles included much higher than the number of components chosen. 

Both the classification and quantification algorithms included error minimization. To evaluate the classification error, we used the percentage of misclassified concentrations. To evaluate the quantification error, we used the root mean square error (RMSE) and the coefficient of determination, R^2^ [51].

## 3. Results and Discussion

### 3.1. Discrimination of Formaldehyde, Formic Acid, and Acetic Acid

All measurements were performed under dry synthetic air (SA) while maintaining the relative humidity (RH) at the lowest possible level to minimise the influence of water vapor on the measurements. This choice allowed us to focus on the three VOCs mentioned, to specifically understand their effects on the sensor signal and to avoid the risk of drawing inaccurate conclusions that might result from cross-sensitivity to RH, which itself induces variations in the signal. In this way, we were able to identify and separate the contributions of the VOCs, RH, and the combination of RH and VOCs, gaining a better understanding of the influence of different gases on the sensor signal, thereby improving our prediction power.

**Figure 3:**
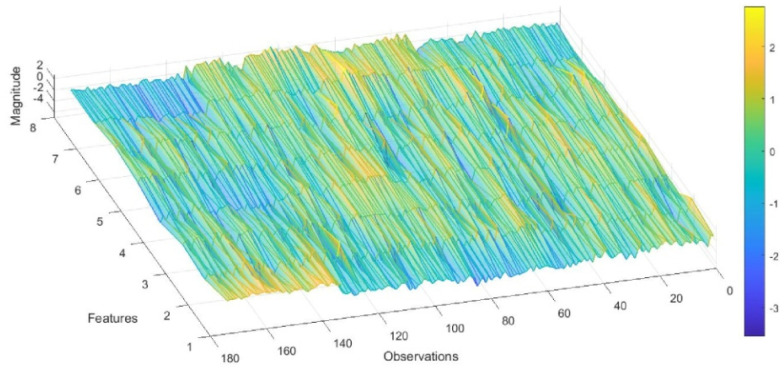
Three-dimensional representation of the parallel coordinates, showing the values of eight features for each observation obtained during the gas measurements.

Eight features were selected from the signals of five virtual sensors when exposed to different concentrations of CH_2_O, CH_2_O_2_, CH_3_COOH, and SA, and were plotted in a three-dimensional parallel coordinate heat map for evaluation (Figure 3). Each of the studied gases contributed to building the model with about 45 observations. Since it is important not just to differentiate between the VOCs but also between the VOCs and SA, we included SA in the study, obtaining a total of about 170 observations. Note that features three to seven contribute with a higher magnitude to the initial observations that relate to CH_2_O. For the following 45 observations that relate to CH_3_COOH, features three to five in the beginning, and then six to eight, show relevance. For CH_2_O_2_, features from three to eight are relevant; meanwhile, for dry air, features from one to three are relevant.

**Figure 4:**
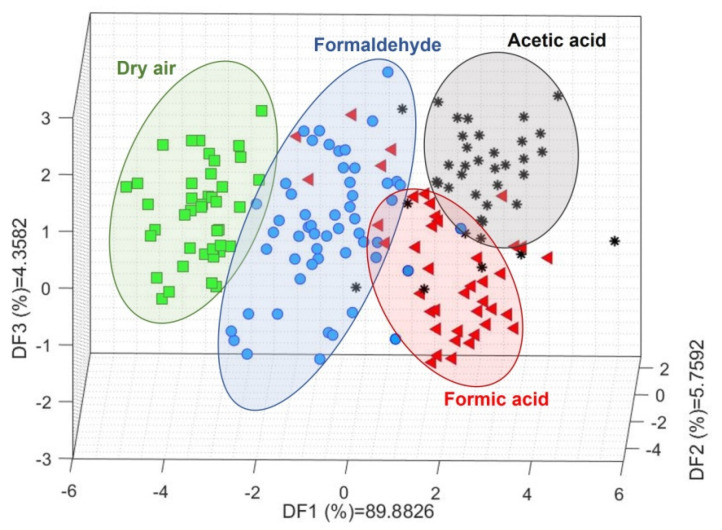
Linear discriminant analysis results for dry air, formaldehyde, formic acid, and acetic acid. The ellipses, as well as the different colours and shapes, are a visual guide to help differentiate each gas.

Linear discriminant analysis (LDA) was first used to develop a visual approach to the discrimination power of the methodology used. The scatter plot resulting from this ML analysis presents a clear trend in the discriminant function (DF) 1 corresponding to the complexity of the molecules studied. From left to right, starting with SA where only oxygen (O_2_) can interact with the sensing material, a distribution from less to more complex VOCs can be observed (Figure 4), i.e., the gases in DF1 are ordered as follows: O_2_, CH_2_O, then CH_2_O_2_ and CH_3_COOH. The difference between CH_2_O_2_ and CH_3_COOH is evidenced by the contributions of DF2 and DF3. In general, CH_2_O_2_ presents central DF2 and negative DF3 values, while CH_3_COOH tends to be located in the positive DF2 and positive DF3 regions.

**Figure 5:**
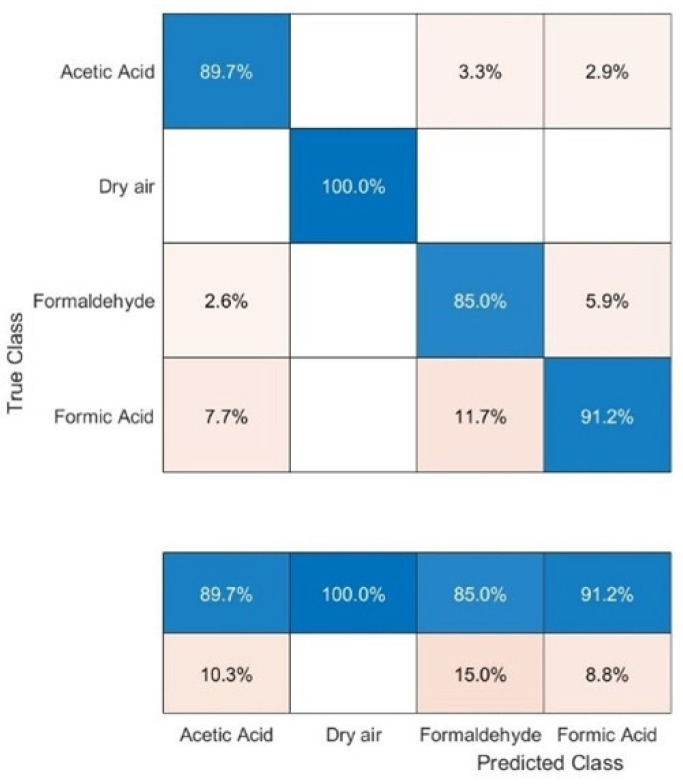
Confusion matrix resulting from the classification performed with a linear 10-fold cross-validated support vector machine model where each group, except for dry air, includes seven different concentrations ranging from 250 to 3000 ppb.

The 10-fold cross-validated (CV) SVM result, represented by a confusion matrix (Figure 5), is used to gain a measurable understanding of the sensor’s ability to distinguish between the studied VOCs. The SVM study reports a validation accuracy of approximately 91%, and classification rates for all the individual gases were always above 85%. We can observe an impeccable classification of 100% for dry air, 91.2% correct classification for CH_2_O_2_, 89.7% for CH_3_COOH, and 85% for CH_2_O.

These results support the feasibility of a simplified method that, as a first approximation, can be used in applications where it is necessary to cost-effectively detect the presence of one or more gases. Thus, the devices and methodology described here as proof-of-concept are, in principle, suitable for selective VOC classification. This possibility shows promise in the use of sensor-based techniques as a suitable alternative to traditional, more expensive, and time-consuming techniques, such as gas chromatography.

### 3.2. Classification of Different Concentrations of Formaldehyde, Formic Acid, and Acetic Acid

**Figure 6:**
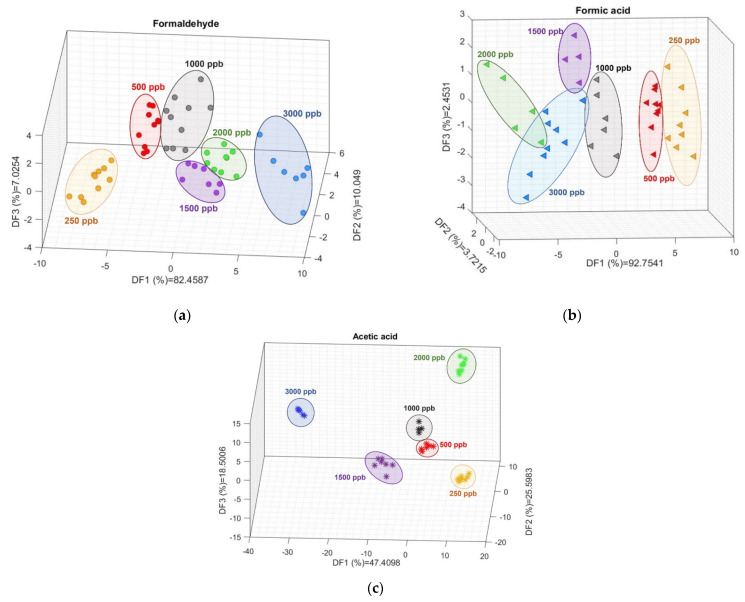
Linear discriminant analysis for (**a**) formaldehyde, (**b**) formic acid, and (**c**) acetic acid. The ellipses, as well as the colors and shapes, are a visual guide to help differentiate each concentration.

The LDA scatter plots for CH_2_O and CH_3_COOH (Figure 6) show clearly separated groups for each concentration studied; although they are less clustered for CH_2_O_2_ than for the other two gases, each concentration presents a clear group in the DF space. For CH_2_O, DF1 shows a distribution from positive to negative values of decreasing concentrations, while DF2 also shows decreasing concentrations from positive to negative values. A similar situation is observed for CH_2_O_2_, except that, in this case, DF1 shows decreasing concentrations from negative to positive values. For CH_3_COOH, a combination of the three DFs is necessary to understand the concentration distribution.

**Figure 7:**
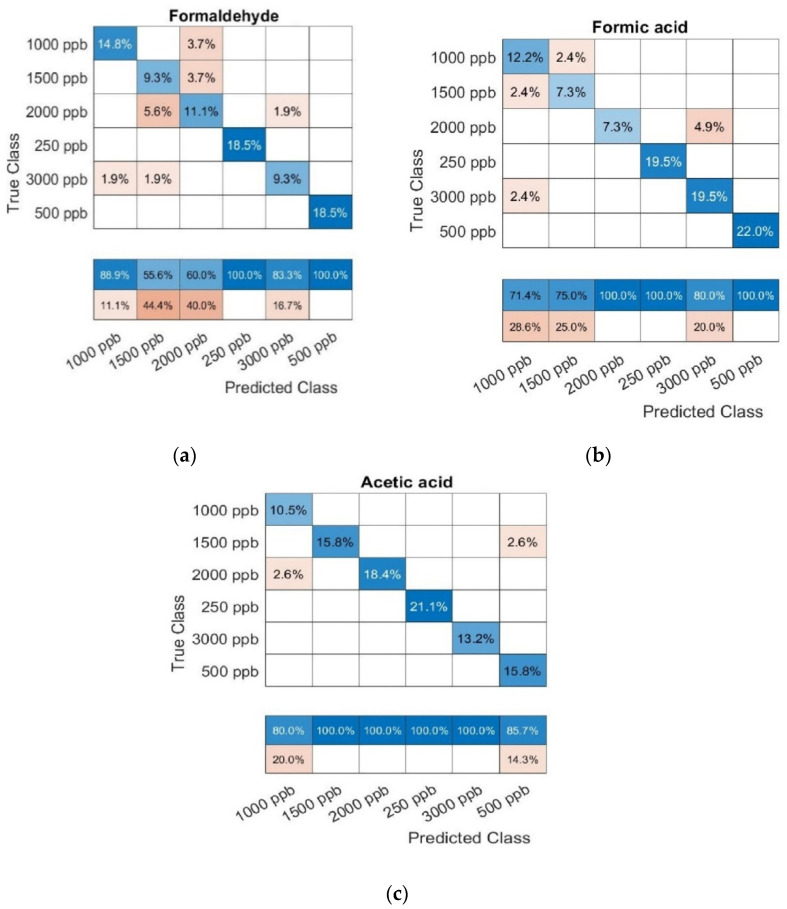
Confusion matrix results from a 10–fold cross–validated support vector machine classification analysis performed for concentrations from 0.25 to 3 ppm for (**a**) formaldehyde, (**b**) formic acid, and (**c**) acetic acid.

The 10-fold CV SVM classification (Figure 7) reports validation accuracies of 81.4, 87.9, and 94.7% for CH_2_O, CH_2_O_2_, and CH_3_COOH, respectively.

These results confirm that our methodology is reliable and appropriate for classifying the VOCs of interest. This opens up the possibility of extensively validating the proposed method with other target gases, and using it in any applications where it is important to discriminate and quantify gas concentrations with a high level of accuracy. In industrial applications, this method can be used to optimise processes or monitor reaction rates. In other applications, such as on-demand controlled ventilation and automated systems, it can be used to reduce energy and power consumption while guaranteeing thermal comfort, safety, and healthy air quality.

### 3.3. Quantification of Formaldehyde, Formic Acid, and Acetic Acid

**Figure 8:**
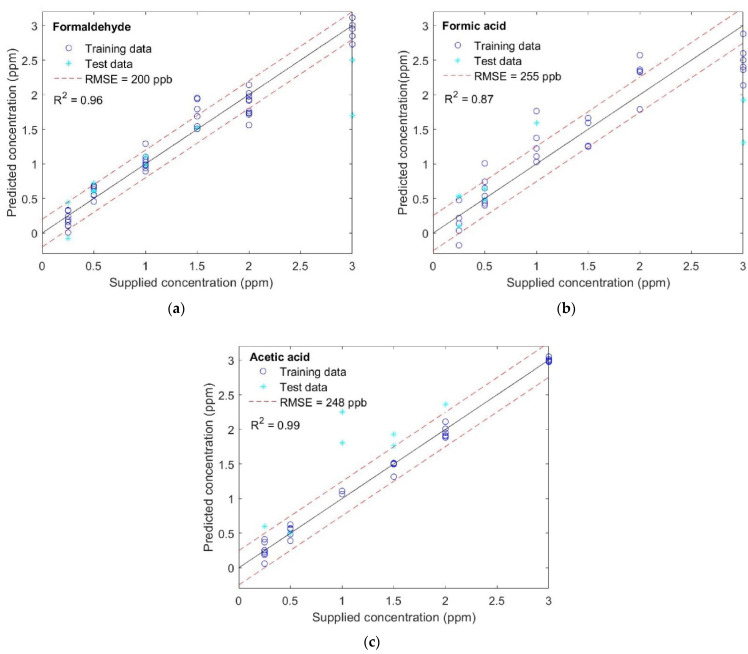
Results of partial least square regression for (**a**) formaldehyde, (**b**) formic acid, and (**c**) acetic acid.

The additional step implemented for better VOC quantification is a 10-fold CV partial least square regression (PLSR) for each of the studied gases (Figure 8). The trained model fits the concentration of the three gases well, with the highest accuracy for CH_2_O, as indicated by the coefficient of determination, R^2^, equal to 0.96 for CH_2_O, 0.87 for CH_2_O_2_, and 0.99 for CH_3_COOH. A possible explanation for this observation concerns the nature of the molecules themselves, and the sensitivity that the gas sensor presents towards these different molecules at this range of temperatures. In our previous study [52], we demonstrated that CH_2_O reports the highest and CH_3_COOH the lowest *relative response* values, due to the differences in molecular mass, both with real and, as in this case, virtual sensors. Therefore, if the chemical gas sensors studied here present higher sensitivity to CH_2_O, it is reasonable to suggest that this improves the detection and quantification of differences in concentrations of this gas. Consequently, the opposite is true of CH_3_COOH. Since a wide concentration range can include a saturation regime or different slopes of the adsorption isotherms, as seems to happen here for concentrations above 2 parts per million (ppm), the accuracy of the models could be enhanced by extending the concentration range studied and performing separated PLSR analysis, focusing on different concentration range regions.

These results further confirm that the methodology implemented here can be used to quantify different VOCs. A continuous concentration scale can be obtained where new and unknown concentration data points, which are not measured directly, can be extrapolated.

### 3.4. Comparison with a Best-in-Class Commercial Gas Sensor

**Figure 9:**
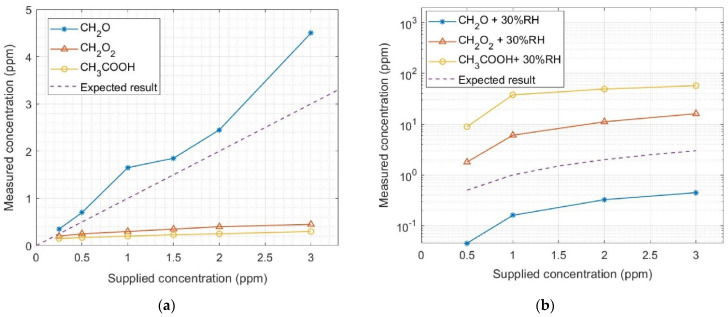
Calibration profile of a commercial TVOC sensor when exposed to different concentrations of formaldehyde (CH2O), formic acid (CH2O2), and acetic acid (CH3COOH), diluted in (**a**) dry synthetic air and (**b**) 30% relative humidity (RH). Note: the results in (**b**) are presented in log scale for a better visualization of the considerable mismatch between the supplied and measured concentrations.

To further demonstrate the pivotal importance of our proposed method, we used a best-in-class low-cost commercial TVOC sensor for laboratory tests under controlled conditions. We verified that the measured gas concentrations obtained with the commercial TVOC sensor (Figure 9a) did not match with the gas concentrations supplied by our gas mixing system when measurements were performed in dry SA (<5% RH). Such a mismatch between the supplied and measured gas concentrations increased considerably when the measurements were performed in humid conditions (Figure 9b), disregarding the possibility of biased results in dry conditions due to lack of water vapor in the ambient. It is worth mentioning that the gas bottles with diluted VOCs, used upon arrival, were certified with stable ppm concentrations for at least one year, and that the MFCs in our gas mixing system were calibrated less than one year before these measurements. These results demonstrate the uncertain reliability of the chemical gas sensors currently available on the market, and the need to find robust methods of improving data quality. Moreover, it is important to underline that the data processing technique used by the commercial sensor is not selective, i.e., it does not identify which gas is being measured. In contrast, our algorithms allow for the classification of the studied VOCs with a 91% success rate. Therefore, the quantification results here presented, obtained with our prototypal chemical gas sensor, can be considered satisfactory and a step forward in the state of the art.

## 4. Conclusions

This study demonstrates that the proper selection and implementation of a sensor’s operation mode, the features to be studied, and machine learning techniques are crucial for overcoming the selectivity issues of chemical gas sensors. We achieved 91% correct classification of three similar VOCs and synthetic air, which was used as the carrier gas. We achieved correct classification between 81 and 95% for different concentrations of these VOCs, and quantification with coefficients of determination (R^2^) between 0.87 and 0.99, depending on the gas species. Therefore, this detailed study of the responses of the gas sensor, when operating in a dynamic mode in synergy with an iterative process of feature extraction, enhances the outcomes both qualitatively and quantitatively, i.e., it optimises discrimination, classification, and quantification. Due to the widespread production, use, and emissions of formaldehyde, formic acid, and acetic acid, our work can potentially benefit many fields and application scenarios. Future work will include an extended characterization of mixtures of VOCs, the influence of RH on the model, and the implementation and application of our algorithms in operational environments.

## Data Availability

The data presented in this study are available on reasonable request from the corresponding author.
